# Larval Frass of *Hermetia illucens* as Organic Fertilizer: Composition and Beneficial Effects on Different Crops

**DOI:** 10.3390/insects15040293

**Published:** 2024-04-20

**Authors:** Giovanni Lomonaco, Antonio Franco, Jeroen De Smet, Carmen Scieuzo, Rosanna Salvia, Patrizia Falabella

**Affiliations:** 1 Department of Sciences, University of Basilicata, Via dell’Ateneo Lucano 10, 85100 Potenza, Italy; giovanni.lomonaco003@unibas.it (G.L.); antonio.franco@unibas.it (A.F.); carmen.scieuzo@unibas.it (C.S.); 2 Spinoff Xflies s.r.l., University of Basilicata, Via dell’Ateneo Lucano 10, 85100 Potenza, Italy; 3 Research Group for Insect Production and Processing, Department of Microbial and Molecular Systems, KU Leuven, 2440 Geel, Belgium; jeroen.desmet@kuleuven.be

**Keywords:** insects, bioconversion, agriculture

## Abstract

**Simple Summary:**

This review explores the potential of black soldier fly larval frass (which is a mixture of insect excrements and leftover substrates) in organic agriculture. Frass can work as a natural fertilizer for agriculture, enriching soil with nutrients and beneficial bacteria. The study investigates how the composition of frass depends on the substrate for larval feed, and how it affects different crops. By categorizing crops and evaluating the impact of frass, the research sheds light on its potential benefits and drawbacks in farming practices. Overall, understanding how black soldier fly frass can enhance soil fertility offers a sustainable solution for agriculture, reducing waste while promoting healthier crop growth.

**Abstract:**

*Hermetia illucens* has received a lot of attention as its larval stage can grow on organic substrates, even those that are decomposing. Black soldier fly breeding provides a variety of valuable products, including frass, a mixture of larval excrements, larval exuviae, and leftover feedstock, that can be used as a fertilizer in agriculture. Organic fertilizers, such as frass, bringing beneficial bacteria and organic materials into the soil, improves its health and fertility. This comprehensive review delves into a comparative analysis of frass derived from larvae fed on different substrates. The composition of micro- and macro-nutrients, pH levels, organic matter content, electrical conductivity, moisture levels, and the proportion of dry matter are under consideration. The effect of different feeding substrates on the presence of potentially beneficial bacteria for plant growth within the frass is also reported. A critical feature examined in this review is the post-application beneficial impacts of frass on crops, highlighting the agricultural benefits and drawbacks of introducing *Hermetia illucens* frass into cultivation operations. One notable feature of this review is the categorization of the crops studied into distinct groups, which is useful to simplify comparisons in future research.

## 1. Introduction

In an era of global climate change and land degradation, ensuring food security for an ever-growing world population of nearly 10 billion people projected in 20–30 years is a significant issue [1]. In this context, fertilizers are important in food production systems [2]. On the one hand, fertilizers can increase agricultural production [3], but on the other hand, they can cause damage to the ecosystem [1,3]. Farmers can adapt fertilization to environmental conditions by replacing chemical fertilizers with organic ones, such as animal waste (e.g., slurry, manure) and crop residues [3]. Another organic fertilizer that has been gaining particular interest in recent years is larval frass (consisting of larval excrements, larval exuviae, and unconsumed feedstock), one of the byproducts of insect breeding systems [4]. Insect farming has many environmental advantages: indeed, if compared with livestock, less land and water are required, and greenhouse gas emissions are lower [5,6]. A particular category of insects is the bioconverter, which can convert large amounts of organic waste into a biomass rich in proteins and fats suitable for animal nutrition [7,8,9,10,11,12]. As one-third of global agricultural and food production is wasted, the possibility of growing insects on former foodstuffs represents a critical solution for reducing food waste by converting it into valuable products relevant for different industrial sectors, including animal feed, human food, cosmetics, pharmaceuticals, and biofuel [13,14,15,16].

**Figure 1:**
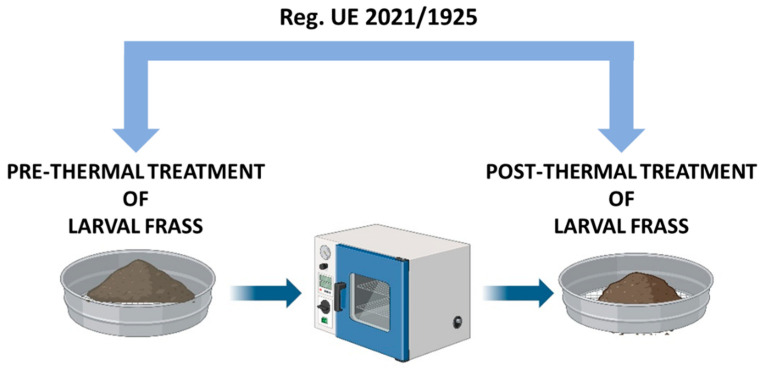
Steps of heat treatment on a small scale. Freshly produced frass were placed in containers and heat-treated in a pre-heated oven for 1 h at 70 °C. After the treatment, the frass can be used in the agricultural field.

The chemical fertilizer sector needs innovative and sustainable production systems to reduce waste and environmental pollution. Considering the speed of the process and the high-value products obtained, the bioconversion of waste by insect larvae has gained significant attention in recent years [17,18]. One of the most promising insect species for bioconversion is *Hermetia illucens* (Diptera: Stratiomyidae), also known as Black Soldier Fly (BSF) [19,20]. Insect bioconversion systems can upcycle a high amount of heterogeneous substrates from organic waste streams into high value proteins and lipids in the form of insect larval biomass, suitable for food and feed. Other products obtained from the insects rearing, in addition to protein and lipids, are antimicrobial peptides [21], chitin, and its derived chitosan [22,23,24]. Larvae can be used as feed to chickens, fish, and pigs because of their high protein content [25,26]. Another application of BSF-derived larval biomass, due to the high amount of lipids, is the production of biodiesel [27]. Chitin and its derivatives due to their numerous applications in several sectors (food, cosmetics, pharmaceuticals, textiles, etc.) have great economic value [28,29,30,31]. Finally, another interesting product deriving from BSF breeding is frass that can be used as fertilizer [32,33]. There is a significant worldwide interest in employing insect larvae to convert organic waste into excellent frass fertilizer using a circular economy strategy [34]. In accordance with the current regulation, in order to meet the microbiological requirements for the use of insect frass as a biofertilizer or soil improver, a heat treatment of 70 °C for 60 min is appropriate (Figure 1) [35]. Indeed, in addition to beneficial microorganisms, there are groups of microorganisms that could pose a problem when utilizing organic fertilizers in agriculture, such as harmful bacteria and fungi; for this reason, several laws ban the use of fertilizers containing *Salmonella* spp., *Escherichia coli*, thermotolerant coliforms, and other microorganisms at specified concentrations on farmed land. In addition, it is extremely important, from an agronomic and financial standpoint, that specific microorganisms are present in organic agriculture fertilizers [36]. It is necessary to identify and understand the most effective heating method for preserving the beneficial microorganism present in insect frass and, at the same time, ensuring that no infections or live larvae are released into the environment [37]. Heating, pelleting, or extrusion of insect frass are examples of possible hygienization procedures, as they may reach the temperature (70 °C) indicated by the European regulation [35]. However, all these procedures require a significant amount of energy input. In order to explore a cheaper and sustainable alternative, the biogas system could be considered a valid technology to be utilized for waste biomass treatment that has a proven sanitization efficacy [38].

### Insect Rearing

The European Commission issued Commission Regulations (EU) 2017/893 and 2021/1372 which authorized the use of insect protein in aquaculture, poultry, and pig feed. This authorization is limited to eight species, including BSF, which must be reared on ‘food quality’ substrates [38]. Processed animal proteins derived from farmed insects may be imported into the EU, provided that the substrate for insect feeding contains only non-animal products or the following animal derivative: fishmeal, blood products of non-ruminants—dicalcium phosphate and tricalcium phosphate of animal origin—hydrolyzed proteins of non-ruminants, hydrolyzed proteins of ruminant hides and skins, gelatin and collagen of non-ruminants, eggs and egg products, milk, milk products, milk products and colostrum, honey, and rendered fats. Furthermore, the substrate for insect feeding and the insects themselves must not be exposed to any other animal-derived components than those listed above. Additionally, the substrate must not contain manure, kitchen garbage, or other animal waste. In addition to these authorized substrates, several studies have been conducted on other types of substrates. It is essential to ensure that the starting material is devoid of dangerous elements and inorganic compounds. Moreover, it is important to reduce the particle size of the material to a diameter of 1–2 cm, since BSF larvae (BSFL) are unable to break down substantial substrate particles, and increasing the surface area of the substrate promotes the proliferation of the related beneficial bacteria [39]. Efficient shredding of diet ingredients also increases the homogeneity of the residue, which improves its quality and simplifies the subsequent separation of larvae and residual material [40]. BSFL can feed on a variety of organic substrates, both vegetable and animal in origin. Under optimal growth conditions (27 °C temperature and 70% humidity), they bioconvert feed into larvae biomass in about 14 days [41,42,43,44]. Since the prospects for rearing BSFL are so promising, many farmers are turning to both the local and large-scale production and breeding of larvae. The rearing process normally entails a lot of human effort, and can thus be perceived as a time-consuming and difficult operation [45]. In addition, insect farming is more environmentally friendly than conventional livestock, requiring less natural resources in terms of land, water, and fertilization [46]. As already mentioned, the main waste product from BSF farming is frass, which can be used in agriculture as an organic fertilizer. This review focuses on frass derived from different substrates, their chemical compositions, thermal treatment, and their applications in agriculture.

## 2. Frass

Insect excrements, known as “frass”, are a significant byproduct of the bioconversion process, consisting of larval excreta, larval exuviae, and unconsumed feedstock. This combination of components yields a substance capable of supplying nutrients and organic matter to soil, modifying soil microbiota, and manipulating plant behavior [47].

Except for BSF and *Tenebrio molitor*, studies on the use of frass from edible and commercial insects as organic fertilizer are currently scarce [48,49,50,51,52,53,54]. Frass has a high potential as a valuable plant biofertilizer (improving soil nutrient content, with a direct effect on plant growth) and/or improving other physical properties of the soil, such as structure, with an indirect effect on plant growth [55,56]. When the majority of the larvae has stopped the feeding period, frass and larval biomass are collected by sieving them [52,57]. Insect frass is gaining popularity as an organic fertilizer due to its high concentration of vital plant nutrients and the presence of chitin, a component of the insect exoskeleton, which seems to stimulate plant defense mechanisms and growth [58]. Since insect farming is expanding as a sustainable alternative element for terrestrial and aquatic animal feed production, and for food waste management, the availability of frass also increases [59]. Frass can be considered a value product to use as biofertilizer, having demonstrating comparable efficacy to organic fertilizers, even when BSFL are fed on various types of substrates.

### 2.1. Frass Composition

Frass is not a uniform product; its quality and composition are heavily influenced by the feed substrates used in the rearing process [52], and also by post-processing, which may be recommended for sanitation reasons [60] and which can significantly alter its properties [61]. Given that BSFL can consume a variety of feeding substrates, the frass composition can likewise vary [47].

Different frass characteristics have a significant impact on plant growth [62].

BSFL actively alter the pH of the substrate towards an alkaline environment; while BSFL may grow at a variety of pH levels, their performance appears to be optimal at higher pH levels of the feeding substrate [63,64,65]. Indeed, Ma et al. [64] found that the best pH value for the BSF production system appears to be slightly acidic (6.0) or somewhat basic (8.0 and 10.0). Adjusting the pH at the beginning of feeding effectively improves larval growth.

The feed substrate initial pH influences the frass pH [53,65]. Ma et al. [64] evaluated that starting from substrates with initial pH values of 6.0, 7.0, 8.0, and 10.0, at the end of the larval feeding phase, frass had pH values ranging from 8.0 to 8.5, while in substrates with initial pH of 2.0 and 4.0, the obtained frass had a pH of 6.0. The reduction in substrate pH caused by BSFL could be attributed to the gut microbes responsible for organic acid generation [64]. Increases in substrate pH, on the other hand, recorded the alkalization of the substrate produced by the release of ammonium ions (NH_4_^+^) and ammonia [66].

Meneguz et al. [65] observed that feeding diets with different pH values (4, 6.1, 7.5, and 9.5) resulted in frass with pH values between 8.9 and 9.4, while Ma et al. [64] recorded frass pH values of 6.0 in larvae reared on diets with pH value 4.

The different pH values could be associated with a different larval density [67]. Indeed, in Meneguz et al. [65], the density was 60 larvae per liter of diet, with 0.96 g of food per larva, while for Ma et al. [64], the density was 100 larvae per liter, with 1.6 g of food per larva. In addition to this hypothesis, the breeding box dimensions, its height, and the amount of provided feed could influence the pH [65]. We could not compare these parameters, since Ma et al. [64] did not report the container dimensions, but only the volume.

Differences in BSF production systems (breeding procedures, feeding substrate, sieving processes, and frass post-processing) can all have an impact on frass DM content, also complicating the comparison of results inter-studies [68].

Optimal conditions for larval growth are 70% of relative humidity and a temperature of 27 °C; at lower temperatures, larvae consume food more slowly [42]. Cheng et al. [69] discovered that although a higher moisture content of food waste results in a faster larval growth rate, it also complicates the separation of the residue from the BSFL biomass. Indeed, the moisture content of the residue is very important in order to facilitate the separation of BSFL biomass. A moisture content between 82% and 86% makes separation difficult and can cause clumping, which can result in subpar sieving results [69,70].

The starting substrate moisture content should be 70–75% for successful sieving of the residue; moreover, separation is better when BSFL are able to reduce the frass moisture content to around 50% [69]. In general, also, the color could be an indicator of optimal sieving property; freshly produced frass has a darker color and a higher moisture content, while dried frass has a lighter color and a lower moisture content.

Gärttling and Schulz [68] averaged the composition of different frass obtained from the larval rearing on different initial substrates (Table 1).

#### 2.1.1. Macronutrients

Macronutrients such as nitrogen (N), phosphorus (P), and potassium (K) are equally vital for plant growth as sunlight, CO_2_, and H_2_O [71]. The macronutrient composition of frass is strongly influenced by the type of food substrate provided to larvae. In Table 2, chemical characteristics (particularly for macronutrients) of frass derived from larvae fed on different feeding substrates are reported.

The larval frass exhibits varying levels of nitrogen, phosphorus, and potassium, depending on the diet fed to the larvae. Frass obtained from feeding larvae with fresh okara yields the highest nitrogen content at 5.1% [76]. The highest percentage of phosphorus (5.2%) was observed in frass obtained by feeding the larvae the Gainesville diet [72], while the highest presence of potassium (4.5%) was detected in frass obtained by feeding the larvae with kitchen waste [78]. The frass obtained by the Gainesville diet showed the better composition in terms of the combined macronutrients nitrogen, phosphorus, and potassium [72]. This diet, together with chicken feed, as highlighted in Table 2, shows its excellent nutritional characteristics for larval growth. Both diets are usually used as controls to evaluate larval growth and to attribute any variations in growth factors solely to the food substrate, although the most widely used, considered as the actual standard diet, is the Gainesville diet. This can be confirmed, as the rearing of BSFL on this diet results in frass with better quality compared to those obtained from larvae fed on the other control diet. Moreover, in Labella et al. [86] investigation, the interest is not only focused on the two tested diets (the Gainesville diet and the 43% sheep whey + 57% seeds diet), but also on the thermal treatment applied to the analyzed larval frass. Indeed, the analyses were performed on frass without any thermal treatment and on the same frass after being treated at 70 °C for 1 h, as reported by the abovementioned European regulations in order to reduce the possible presence of dangerous bacteria/pathogens.

#### 2.1.2. Micronutrients

For the optimal growth of the plant, in addition to macronutrients and also micronutrients, including calcium (Ca), magnesium (Mg), sodium (Na), iron (Fe), copper (Cu), manganese (Mn), and zinc (Zn), are fundamental. Table 3 shows the micronutrient composition of the different frass obtained from larvae reared on different feeding substrates.

As can be seen from the values reported in Table 3, the missing analysis for some micronutrients in literature leads to a less accurate analysis than for macronutrients. Many authors do not determine the presence of sodium in the analysis of frass, but knowing the amount of sodium is important to understand for which crops and which type of soil frass would be useful. For instance, when the concentration of sodium in the substrate increases, its concentration also increases in plant tissue, reducing plant fitness, particularly in plants that are very susceptible to salt stress [89,90].

### 2.2. Microbiological Composition of BSFL Frass

Microorganisms included in organic plant fertilizers can have several benefits [36]: they can boost nutrient usage, promote plant development, tolerance to abiotic stress, and resistance to diseases and pest attacks [36,49,91]. The use of beneficial microorganisms in agriculture [92] is an essential alternative method for supplying healthy food in a sustainable way by minimizing the use of chemical fertilizers, pesticides, and herbicides [93,94]. Among the interesting microorganisms, genera such as *Azospirillum, Bacillus*, *Mycorrhizae*, *Pseudomonas*, *Rhizobia*, *Streptomyces*, *Trichoderma*, and *Bacillus* species are the most encouraged for their easy production and application among these regularly utilized microbes [95]. Insects have a rich biodiversity of microorganisms (protists, fungi, archaea, and bacteria) in their gut that play critical roles in several aspects of insect physiology [96,97]. The microbiome dynamics of BSFL are of particular interest in order to gain insight into the physiology of the insect and, furthermore, to boost the yield of the rearing or conversion process [98]. The composition of BSFL gut microbiota varies according to the feeding substrate [98] and other breeding variables, such as the temperature [99]. Yue et al. [100] fed BSFL on chicken and pig manure, and they found 10 genera persisting in all samples: *Enterococcus* (average relative abundance of 24.1%), *Providencia* (21.7%), *Morganella* (14.5%), *Klebsiella* (4.9%), *Ignatzschineria* (3.3%), *Clostridium* (2.4%), unclassified *Enterobacteriaceae* (1.8%), *Actinomyces* (1.6%), *Proteus* (1.1%), and *Vagococcus* (0.6%). Gold et al. [101] identified several bacteria characteristic of the gut and BSFL frass, including the *Firmicutes*, *Proteobacteria*, *Bacteroidetes* phyla, and *Dysgonomonas*, *Morganella*, *Providencia*, *Proteus*, *Sphingobacterium*, *Pseudomonas*, and *Bacillus* genera. Raimondi et al. [101] determined the microbial composition of frass obtained from BSFL reared on a diet composed of 25% maize meal (milling waste), 15% wheat bran, 10% alpha meal, and 50% water. The microbial composition was represented by *Proteobacteria*, *Actinobacteria*, *Firmicutes*, and *Enterobacteriaceae.* Another example is reported by Fuhrmann et al. [82], in which BSFL were fed on spent malted barley grain, and the frass was used as a soil conditioner for growing clover grass, affecting the soil microbial population, which increased microbial activity (basal respiration) and crop output. The most abundant phyla found in that study were the groups of *Proteobacteria* (31%), *Actinobacteria* (24%), *Bacteroidetes* (15%), *Firmicutes* (15%), and *Chloroflexi* (11%).

#### Impact of BSFL Frass Microorganisms

In addition to macro- and micro-nutrients and micro-organisms, frass can also have an impact on soil micro-organisms [102,103]. Nurfikari [104] fed BSFL on agro-industrial waste, and the resulting frass stimulated soil bacteria and fungi such as *Bacilli*, *Actinobacteria*, *Gammaproteobacteria*, and *Mortierellomycetes*, as well as bacterial chitinase genes, which are known to suppress some pathogens. Concerning the plant’s defense against pathogens, the addition of frass to the soil is a viable treatment for *Fusarium* wilt in lettuce. Chitin-rich exuviae absorption into soil has had even more positive benefits on disease control than only frass. Another interesting study showed that the use of frass in a bean crop reduced plant death due to *Fusarium oxysporum* [105]; in particular, frass obtained by feeding BSFL on the Gainesville diet exhibits antifungal activity against *F. oxysporum* [79]. Gebremikael et al. [106] demonstrated that the addition of frass reduced the presence of a soil pathogen *Rhizoctonia solani*. In tests on beans (*Phaseolus vulgaris*) infected with this pathogen, the application of frass resulted in a 50% reduction in the disease rate, which was attributed to chitinase activity [106]. Another study examined the extract of filtered and unfiltered frass deriving from larvae fed on two distinct diets: the Gainesville diet, and a diet consisting of a mix of fruits, vegetables, bakery, and beer waste (FVBB). The extract of frass from the unfiltered Gainesville diet inhibited the mycelia *Alternaria solani*, *Botrytis cinerea*, *F. oxysporum*, *Pythium capsici*, *R. solani*, and *Sclerotinia sclerotiorum*. In contrast, the frass-derived extract from the FVBB diet inhibited only *B. cinerea*, *S. sclerotiorum*, and, to a lesser extent, *A. solani*. Filtered extracts of both fractions revealed no change in micellar growth [79]. The filtration probably resulted in the loss of inhibitory agents for mycelial development.

## 3. Frass Application

Several works analyze the application of frass as a fertilizer for plants. The effects of frass application depend on frass composition, dosage, and plant species. Below, the larval frass effects on different crops are described and reported in Table 4 and Table 5. The two tables are divided by plant family: Table 4 for Gramineae, and Table 5 for Asteraceae, Cruciferous, Lamiaceae, Plantaginaceae, and Solanaceae. However, studies conducted on species belonging to the families reported in Table 5 are limited compared to those on Gramineae. In each Table, the initial feeding substrate of the BSFL, the dosage that gave the best growth response, the duration of plant growth, the plant species tested, and the effects of the optimum dose on the plant are reported.

Mason [109] tested frass obtained from the bioconversion process of fruit/vegetable pulp + poultry litter on barley crops (*Hordeum vulgare*). The two doses of frass tested were 4% and 8%. The growth performances of the plants were evaluated at germination and after 44 days, and biomass yield and chlorophyll content were evaluated. *Hordeum vulgare* plants administered with 4% frass showed an increase in shoot length, biomass, and photosynthetic activity. Carroll et al. [109] assessed the effects of frass derived from feeding BSFL with spent grain on four cereals: oats (*Avena sativa* cv. *Apollon*), spelt (*Triticum spelta*), triticale (*Triticosecale* cv. *Trisem*), and barley (*Hordeum vulgare* cvs. *Westminster* and *Quadriga*), all belonging to the Gramineae family. The goal was to determine the optimal frass dosage that would enhance plant growth. The doses of frass tested were 1, 2, 4, 8, 12, and 16 g per pot. The optimal frass dose for each plant species was between 6 and 8 g; the exact value was determined on the basis of the fitted polynomial curves. The ideal frass dosage varied among the tested cereal species: 7.1 g for oats, 6.3 g for spelt, 6.8 g for triticale, and 7.8 g for barley. Beesigamukama et al. [54] tested the effect of frass obtained by feeding BSFL with spent grain on maize variety H513. This trial was conducted in the open field, so frass was distributed on the soil surface. The doses used were: 2.5 t/ha, 5.0 t/ha, and 7.5 t/ha. The 2.5 t/ha of frass resulted in an increase in plant height and higher chlorophyll content. Wang et al. [108] fed BSFL on chicken manure, and the frass obtained at the end of the bioconversion process was tested on rice crops (*Oryza sativa*). The test was conducted in a bucket by homogeneously mixing soil and BSFL frass. The doses of frass applied were 2%, 4%, 6%, and 8% by weight to the soil. The production yield in the first and in the second year of frass application were evaluated. The increase in yield in the first year of application corresponded to the 4% dose by weight, while the 8% was considered almost lethal as it led to a reduction in yield; in the second year, a better yield was observed with the 8% dose. Therefore, the frass may have a fertilizing effect later in the year of application, and, in this particular case, provide nutrients for rice growth in the second year. In another study carried out by Menino et al. [53], the frass obtained by feeding BSFL with onion and potato waste was tested on *Lolium multiflorum*. The doses of frass used for the test were: 25%, 50%, 75%, 100%, 125%, and 150% of the total nitrogen requirement. The total nitrogen requirement was 140 kg/ha for the tested crop. The trial was conducted in pots with a sand and soil content ratio of 1:2, and the weight was 3.6 kg. For the whole production of *Lolium multiflorum*, a linear increase in biomass was observed up to the 100% treatment (equivalent to 140 Kg/ha). With regard to the soil, an increase in organic matter, phosphorus, and potassium was observed at the end of the trial, especially for the higher-dose treatments. The application of frass on species belonging to the Gramineae family in general resulted in a greater increase in height and biomass in the right dose. In Labella et al. [86], the frass (thermally and not thermally treated) extract, obtained by feeding larvae with the Gainesville diet and the 43% sheep whey + 57% seed diet, was tested in different dilution percentage; for both diets, 25% diluted frass extract showed on barley an increase in chlorophyll content and root dry weight. As shown in Table 4, the optimal dose of frass to be applied in order to have beneficial effects is highly variable in relation to the starting feeding substrate of the BSFL and the chosen cultivar.

Setti et al. [72] tested the frass obtained by feeding BSFL with the Gainesville diet, on Solanaceae (*Solanum lycopersicum*), Labiate (*Ocimum basilicum*), and Asteraceae (*Lactuca sativa*). The tests were conducted in pots, and the different doses of frass applied were: 10%, 20%, 30%, and 40%. The optimal dose that led to an increase in plant growth was 10–20% by volume, showing agronomic properties comparable to chemical fertilizer. The results obtained by Setti et al. [72] were consistent with previous studies, which stated that partially substituting peat with agro-industrial byproducts results in an increase in plant biomass [115,116,117,118]. Organic fertilizers can replace chemical ones as they can provide, in addition to nutrients, microorganisms that stimulate crop growth [119]. Furthermore, Setti et al. [72] deduced that a frass dosage of up to the 20% added to peat resulted in a favorable response in crop growth. An important aspect of their findings is the potential reduction in peat application and inorganic fertilizer.

Radzikowska-Kujawska et al. [114] used BSFL frass on *Ocimum basilicum*, with two doses (10 g/L and 12.5 g/L). The plants were subjected to two conditions: ideal irrigation conditions, and drought, the latter being an important condition to evaluate, as fertilizers can be effectively used to improve plants stressed by water scarcity. Frass showed enhanced fresh weight and photosynthetic activity in both the analyzed conditions.

Hodge and Conway [111] tested different doses (0.5, 1, 2, 4, 6, 8, and 10 g per pot) of frass, obtained from larvae reared on spent grain, on two plant species: Plantaginaceae (*Plantago lanceolata* L.), and Asteraceae (*Cichorium intybus* L.). The optimal dose (4 g per pot) effectively improved plant development. These forage species may not respond well to higher levels of frass application, particularly in soils with a high plant nutrient content or organic matter, or when the plants do not exhibit major nutrient shortages. Indeed, they detected a higher plant mortality at dosages superior to 4 g of frass.

Based on the different studies reported in literature, a direct comparison to evaluate which combination of factors results in the frass with the best characteristics is difficult, since frass are obtained from BSFL feed on different substrates coupled with variations in the plant species. Moreover, there is no homogeneity in the units of measurement of frass used; some researchers apply frass by expressing doses in weight (g), while others apply frass doses by expressing them in volume (%). Moreover, the duration of the trials on the different plant species is a further parameter that makes the comparison even more difficult. For example, the duration time ranges from a minimum of 30 days for lettuce [72] to a maximum of 180 days to assess the grain yield of rice [108].

## 4. Conclusions

The aim of this review is to compare frass obtained by feeding BSFL with different feeding substrates. The comparison among the different frass was focused on the composition in macro and micronutrients useful for plants. The concentration of these nutrients was found to be highly variable in relation to the type of feeding substrate provided to the larvae. Knowing the chemical composition of the frass obtained by feeding BSFL with different substrates could make it possible to understand, on the basis of the nutritional requirements, to which plant species it is best to apply frass with specific characteristics. Another important aspect explored in this review concerned the determination of the optimal dose of frass to trigger a positive response in certain plant parameters. We reported and critically analyzed the results obtained by applying frass produced from different feeding substrates for BSFL and different plant species on which BSFL frass was tested. However, in some cases, where the feeding substrate for BSFL was the same, the plant species on which the frass was tested was different, complicating any comparison. In conclusion, it is possible to state that several studies show that BSFL frass, when applied in the optimal doses, which depends on the selected plant culture, has beneficial effects on plant growth; meanwhile, when applied at high doses, it has a suppressive or deleterious effect.

In addition, BSFL residues, with high plant-nutritional value and microbial capabilities to promote plant growth, have the potential to improve sustainable agricultural production, particularly in low-income countries where BSFL rearing is a promising low-threshold technology in the circular economy.

## 5. Future Perspective

Given the enormous interest in frass in recent years and its potential significance in agriculture as a fertilizer and soil conditioner (how much it has good plant nutrient content) or just as a soil conditioner (in case of low nutrient content), it would be interesting evaluate the whole microbiological component present in frass and how this varies in relation to the starting substrate provided to BSFL, as reported for nutrients. Due to the limited available literature, another interesting aspect to investigate is the impact of heat treatment at 70 °C for 1 h on both the nutrient and microorganism content of frass. Additionally, a promising avenue for future work could be to evaluate the influence of frass on soil structure and stability. In order to get a comprehensive understanding of the potential of frass, it is advisable to investigate all these aspects collectively rather than individually. Future research is needed to further elucidate these correlations.

## Figures and Tables

**Table 1:** Average composition of different frass obtained from different initial substrates. Data are reported on dry matter (DM) values [68].

Physical Parameters	
Dry matter (DM)	69.6%
Organic matter (OM)	86.2% DM
pH	7.46
C:N ratio	14.7
Electrical conductivity (EC)	4.0 mS cm^−1^
Macronutrients	
Total nitrogen (Nt)	32.2 g kg^−1^ DM
Ammonium nitrogen (NH_4_^+^-N)	5.6 g kg^−1^ DM
Phosphorus (P)	12.4 g kg^−1^ DM
Potassium (K)	29.3 g kg^−1^ DM
Micronutrients	
Magnesium (Mg)	4.7 g kg^−1^ DM
Sodium (Na)	5.7 g kg^−1^ DM
Calcium (Ca)	8.8 g kg^−1^ DM
Sulfur (S)	6.3 g kg^−1^ DM
Copper (Cu)	43.8 mg kg^−1^ DM
Boron (B)	34.5 mg kg^−1^ DM
Zinc (Zn)	136.3 mg kg^−1^ DM
Iron (Fe)	1808.4 mg kg^−1^ DM
Manganese (Mn)	79.5 mg kg^−1^ DM

**Table 2:** Macronutrients composition of BSFL frass deriving from different initial substrates.

Feeding Substrate		Macronutrient (%)			References
	C	N	P	K	
Gainesville diet *	35.2	3.8	5.2	4.1	[72]
Distiller grains *	–	3.4	0.8	1.1	[73]
Brewery spent grain ****	38.6	3.6	0.5	0.3	[61]
Okara and wheat bran ***	37.1	4.8	1.0	0.9	[62]
Okara and wheat bran ******	30.6	3.2	0.8	0.5	[62]
Household waste *	35.8	2.2	0.5	0.7	[74]
Wheat bran *	35.7	2.8	1.4	2.3	[75]
Brewery spent grain ****	35.2	2.1	1.2	0.2	[54]
Fresh okara *	37.1	5.1	0.3	1.9	[76]
Chicken manure *	23.6	2.3	1.1	1.8	[77]
Pig manure *	26.8	2.4	2.1	1.0	[77]
Chicken feed *	47.9	2.6	–	–	[52]
Grass cuttings *	44.3	2.4	–	–	[52]
Fruits and vegetables *	48.8	1.8	–	–	[52]
Cow manure *	27.7	1.9	1.0	0.2	[77]
Vegetables *	38.7	2.8	1.5	3.3	[53]
Kitchen waste *	-	3.3	3.1	4.5	[78]
Gainesville diet *	50.8	2.0	1.9	3.7	[79]
Fruit/vegetable/bakery/brewery *	51.4	2.7	1.3	2.9	[79]
Ratios of water hyacinth, fruit waste, and manure (65:25:10) *	23.5	0.8	0.4	2.6	[80]
Ratios of water hyacinth, fruit waste, and manure (50:40:10) *	24.8	1.4	0.4	2.5	[80]
Ratios of water hyacinth, fruit waste, and manure (35:55:10) *	25.3	1.6	0.3	2.2	[80]
Ratios of water hyacinth, fruit waste, and manure (15:75:10) *	27.0	1.7	0.3	1.7	[80]
Ratios of water hyacinth, fruit waste, and manure (10:80:10) *	29.1	1.9	0.2	0.2	[80]
Food waste, chicken feces, and sawdust (3:2:1 ratio) *	-	1.7	1.1	2.1	[81]
Maize straw *	-	0.6	2.5	2.1	[57]
Spent malted barley grain *	40.1	3.2	0.6	0.3	[82]
Hemp waste *	-	2.0	0.7	1.5	[83]
Mix grains, fruits, and vegetables *****	-	-	1.4	1.8	[84]
Food waste *	-	1.5	0.9	1.1	[85]
Gainesville diet **	51.5	1.3	0.9	1.9	[86]
Gainesville diet ***	51.1	1.9	0.9	1.9	[86]
43% sheep whey + 57% seeds **	54.3	1.6	0.5	1.1	[86]
43% sheep whey + 57% seeds ***	54.3	1.9	0.5	1.2	[86]
Gainesville diet *	24.0	1.5	1.2	2.1	[87]
Gainesville diet *	-	-	0.9	2.9	[88]

Asterisks refer to treatment of frass derived from larval rearing on the specific substrate: * not available; ** with thermal treatment at 70 °C for 1 h in according to Reg UE 2021/1925; *** without thermal treatment; **** composted for 5 weeks; ***** treated at 100° C for 48 h; ****** aerated compost.

**Table 3:** Micronutrient composition of BSFL frass deriving from different initial substrates.

Feeding Substrate	Micronutrients							References
	Ca	Mg	Na	Fe	Cu	Mn	Zn	
	(g kg^−1^)			(mg kg^−1^)				
Gainesville diet *	45	8.0	3.0	600	46.1	−	140	[72]
Distillers grains *	13	3.0	5.0	125	15	45	90	[73]
Brewery spent grain ****	9.7	1.0	–	310	25	109	182	[61]
Okara and wheat bran ***	1.3	0.1	–	26	2.2	4.2	0.1	[62]
Okara and wheat bran ******	0.8	0.2	–	26	0.7	2.3	0.1	[62]
Household waste *	10	0.9	0.8	240	10	10	10	[74]
Wheat bran *	–	0.3	–	15	8.9	19.4	15	[75]
Brewery spent grain ****	0.2	0.2	–	–	–	–	–	[54]
Fresh okara *	16.8	10.5	–	3.7	0.9	0.2	1.7	[76]
Vegetables *	15	7.0	0.3	896	19	149	137	[53]
Fruit/vegetable/bakery/brewery *	0.4	4.2	9.7	150.5	9.0	17.0	57.3	[79]
Ratios of water hyacinth, fruit waste, and manure (65:25:10) *	1.4	1.9	-	537	42.9	55.7	78.2	[80]
Ratios of water hyacinth, fruit waste, and manure (50:40:10) *	1.4	1.5	-	534.3	36.6	33.7	68.3	[80]
Ratios of water hyacinth, fruit waste, and manure (35:55:10) *	0.9	1.1	-	415.7	34.5	32.0	60.2	[80]
Ratios of water hyacinth, fruit waste, and manure (15:75:10) *	0.6	0.9	-	95.0	32.6	30.9	43.3	[80]
Ratios of water hyacinth, fruit waste, and manure (10:80:10) *	0.3	0.7	-	290.3	22.7	21.5	38.0	[80]
Spent malted barley grain *	6.4	2.2	0.2	4100	12.8	100	100	[82]
Hemp waste *	19.2	4.6	7.3	1111	26.1	163.9	187.5	[83]
Mix grains, fruits, and vegetables *****	18.1	3.8	6.2	-	44	76	372	[84]
Food waste *	-	-	-	-	57	-	206	[85]
Gainesville diet **	2.1	6.8	0.9	-	-	-	89.3	[86]
Gainesville diet ***	4.4	7.2	0.9	-	-	-	93.2	[86]
43% sheep whey + 57% seeds **	1.1	3.7	1.9	-	-	-	50.1	[86]
43% sheep whey + 57% seeds ***	1.3	3.8	2.0	-	-	-	55.2	[86]
Gainesville diet *	5.0	5.0	-	-	-	-	-	[87]
Gainesville diet *	8.9	5.2	0.9	507	9.8	66.0	58.2	[88]

Asterisks refer to treatment of frass derived from larval rearing on the specific substrate: * not available; ** with thermal treatment at 70 °C for 1 h in according to Reg UE 2021/1925; *** without thermal treatment; **** composted for 5 weeks. ***** treated at 100 °C for 48 h; ****** aerated compost.

**Table 4:** Larval growth substrate, optimal frass dosage, experiment duration, effect of frass doses and plant species of Gramineae family.

Larval Growth Substrate	Optimal Frass Dosage	Plant Species	Experiment Duration	Effect of Frass Doses	Reference
Fruit/vegetable pulp + poultry litter *	4% by volume	*Hordeum vulgare*	44 days	Increased shoot length, increased biomass, and increased photosynthetic activity	[107]
Spent grain ****	2.5 t/ha	* Zea mays *	125 days	Increased plant height and chlorophyll content	[54]
Chicken manure *	4% by weight first year	*Oryza sativa*	About 180 days	Increased productive yield	[108]
Chicken manure *	8% by weight second year	*Oryza sativa*	About 180 days	Increased productive yield	[108]
Spent grain *	7.1 g for plot	*Avena sativa* cv. *Apollon*	About 42–49 days	Increased shoot growth	[109]
Spent grain *	6.3 g for plot	*Triticum spelta*	About 42–49 days	Increased shoot growth	[109]
Spent grain *	6.8 g for plot	*Triticosecale* cv. *Trisem*	About 42–49 days	Increased shoot growth	[109]
Spent grain *	7.8 g for plot	*Hordeum vulgare* cv. *Westminster*	About 42–49 days	Increased shoot growth	[109]
Spent grain *	7.8 g for plot	*Hordeum vulgare* cv. *Quadriga*	About 42–49 days	Increased shoot growth	[109]
Onion and potato waste *	100% of nitrogen demand	*Lolium multiflorum*	About 210 days	Increased production	[53]
Gainesville diet **	frass extracts 25%	* Hordeum vulgare *	until the first leaf was produced.	Increased root dry weight and chlorophyll content	[86]
Gainesville diet ***	frass extracts 25%	* Hordeum vulgare *	until the first leaf was produced.	Increased root dry weight and chlorophyll content	[86]
43% sheep whey + 57% seeds **	frass extracts 25%	* Hordeum vulgare *	until the first leaf was produced.	Increased root dry weight and chlorophyll content	[86]
43% sheep whey + 57% seeds ***	frass extracts 25%	* Hordeum vulgare *	until the first leaf was produced.	Increased root dry weight and chlorophyll content	[86]

Asterisks refer to treatment of frass derived from larval rearing on the specific substrate: * not available; ** with thermal treatment at 70 °C for 1 h in according to Reg UE 2021/1925; *** without thermal treatment; **** compostated for 5 weeks.

**Table 5:** Larval growth substrate, optimal frass dosage, experiment duration, effect of frass doses and plant species of different families (Asteraceae, Brassicaceae, Solanaceae, Plantaginaceae, and Labiate).

Larval Growth Substrate	Optimal Frass Dosage	Plant Species	Experiment Duration	Effect of Frass Doses	Reference
	Asteraceae				
Okara *	10% by volume	* Lactuca sativa *	45 days	Development similar to control	[76]
Gainesville Diet *	10–20% by volume	* Lactuca sativa *	30 days	Increased plant growth (height, stem diameter, leaf number)	[72]
Household waste *	30 t/ha	* Lactuca sativa *	42 days	Increased productive yield	[110]
Spent grain *	4 g per pot	*Cichorium intybus* L.	49 days	Increased in DM shoot	[111]
	Brassicaceae				
Household food waste **	0.37 kg/m^3^	*Brassica oleracea var. sabellica*	35 days	Increased plant weight and height	[112]
Organic waste *	15% of the weight	*Brassica rapa* L.	35 days	Increased fresh plant weight	[113]
	Plantaginaceae				
Spent grain *	4 g per pot	*Plantago lanceolata* L.	49 days	Increased in DM shoot	[111]
	Labiate				
Unspecified *	10 g/L	*Ocimum basilicum*	46 days	Increased fresh weight and photosynthetic activity	[114]
Gainesville Diet *	10–20% by volume	*Ocimum basilicum*	30 days	Increased plant growth (height, stem diameter, leaf number)	[72]
	Solanaceae				
Gainesville Diet *	10–20% by volume	*Solanum lycopersicum*	30 days	Increased plant growth (height, stem diameter, leaf number)	[72]

Asterisks refer to treatment of frass derived from larval rearing on the specific substrate: * not available; ** treated at 100 °C for 48 h.

## Data Availability

The data presented in this study are available in the cited articles.
